# The Phenotype-Fitness Map in Experimental Evolution of Phages

**DOI:** 10.1371/journal.pone.0027796

**Published:** 2011-11-22

**Authors:** James J. Bull, Richard H. Heineman, Claus O. Wilke

**Affiliations:** 1 The Institute for Cellular and Molecular Biology, Center for Computational Biology and Bioinformatics, Section of Integrative Biology, The University of Texas at Austin, Austin, Texas, United States of America; 2 Section of Integrative Biology, The University of Texas at Austin, Austin, Texas, United States of America; 3 The Institute for Cellular and Molecular Biology, Center for Computational Biology and Bioinformatics, Section of Integrative Biology, The University of Texas at Austin, Austin, Texas, United States of America; Michigan State University, United States of America

## Abstract

Evolutionary biologists commonly interpret adaptations of organisms by reference to a phenotype-fitness map, a model of how different states of a phenotype affect fitness. Notwithstanding the popularity of this approach, it remains difficult to directly test these mappings, both because the map often describes only a small subset of phenotypes contributing to total fitness and because direct measures of fitness are difficult to obtain and compare to the map. Both limitations can be overcome for bacterial viruses (phages) grown in the experimental condition of unlimited hosts. A complete accounting of fitness requires 3 easily measured phenotypes, and total fitness is also directly measurable for arbitrary genotypes. Yet despite the presumed transparency of this system, directly estimated fitnesses often differ from fitnesses calculated from the phenotype-fitness map. This study attempts to resolve these discrepancies, both by developing a more exact analytical phenotype-fitness map and by exploring the empirical foundations of direct fitness estimates. We derive an equation (the phenotype-fitness map) for exponential phage growth that allows an arbitrary distribution of lysis times and burst sizes. We also show that direct estimates of fitness are, in many cases, plausibly in error because the population has not attained stable age distribution and thus violates the model underlying the phenotype-fitness map. In conjunction with data provided here, the new understanding appears to resolve a discrepancy between the reported fitness of phage T7 and the substantially lower value calculated from its phenotype-fitness map.

## Introduction

Fitness is the ultimate metric of natural selection. Our understanding and appreciation of how fitness is determined relies on partitioning an organism into components known as phenotypes, such as fecundity, survival, behavior, physiology, and many others. A phenotype-fitness map is the specific relationship between fitness and the phenotype states of individuals (e.g., a particular beak size or number of offspring). From these relationships, we can anticipate how the phenotypes will evolve under natural selection.

It is often pragmatic, if not necessary, to limit phenotype-fitness maps to just a few of the phenotypes determining total fitness. Inferences of evolution based on these disembodied phenotypes should be robust if the variation affecting those phenotypes does not have pleiotropic effects outside those phenotypes or affect trade-offs involving other phenotypes. Yet some model systems enable the measurement of a set of phenotypes that completely determines fitness, and these systems should be of special importance in understanding the strengths and weaknesses of approaches that use phenotypes isolated from the whole. One such model is the growth of a bacterial virus (bacteriophage or ‘phage’) on a continual excess of hosts. In this system, phage fitness is thought to be completely determined by a mere three phenotypes, all easily measurable [1] and translatable into total fitness [2]–[6]. In addition, the system allows independent determination of phage fitness as an empirical check on the complete phenotype-fitness map. This system thus seems well suited to explore the strengths and weaknesses of phenotype-fitness maps as a general tool in evolutionary biology.

Yet despite its simplicity, the currently understood phage phenotype-fitness map does not match reality. In the few cases that a comparison is possible between direct measures of fitness and fitness calculated from the full set of phenotypes, there are serious discrepancies [3], [7], [8] (and see below). Given the importance of phenotype-fitness maps in evolutionary biology and the accessibility of this unique system to empirical and analytical exploration, it seems justified to identify reasons for the incongruities, which we attempt here.

### Growth rate as phage fitness: biology and mechanics

Bacteriophages are viruses that reproduce by infecting and killing bacteria. Under suitable growth conditions, they typically multiply much faster than bacteria and can quickly exhaust the host population. In the lab, however, they can be propagated by dilution and serial transfer to new cultures of hosts so that the phage population is undergoing continual expansion without ever overwhelming the host population. In this environment, the relevant measure of phage fitness is growth rate of its population. In turn, growth rate is determined by host density and three phage parameters: burst size (fecundity), lysis time (time between infection and cell bursting to release progeny), and adsorption rate. When using large numbers of cells and phage, the process is appropriately modeled by mass action dynamics [1], [2], [5] unless one is concerned with the fate of rare mutants [6]. Furthermore, all 3 phage parameters can be measured in a single assay [1].

Under serial transfer, growth of the culture eventually becomes emancipated from initial conditions, and an approximate formula for growth rate of the phage population is trivially 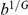, where 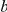 is burst size and 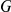 is average generation time [2], [4]. Average generation time is determined from lysis time, adsorption rate, and cell density. Unfortunately, use of the average generation time is known to underestimate actual phage growth rate, seriously so when cell density is low [9]. A more explicit formula has overcome this bias [5], [6], reviewed below, although even that model makes the unrealistic assumption that the phage parameters do not vary.

### Incongruity between the phenotype-fitness map and direct measures

In a study of phage T7, observed fitness of a genotype adapted to lab conditions was nearly 48 doublings/hr [8], whereas the calculated fitness is 39 (using the formula of [5]). We have experienced other discrepancies of 3–5 doublings/hr (unpublished and [10]). Wang [7] and Shao and Wang [3] have also observed discrepancies, although their method of propagation does not lend itself easily to the analytic formula. These are major discrepancies, especially when considering that doublings/hr is an exponential measure of fitness, whereby even small differences have large effects on population behavior. These incongruities motivate this paper.

It may seem that any discrepancy between a direct measure of fitness and a measure calculated from a phenotype-fitness map must result from a flawed map instead of a flawed direct measure. Despite this intuition, both can be at fault. The phenotype-fitness map can predict the wrong fitness either because the phenotypes are measured incorrectly or because they are converted into fitness incorrectly. Alternatively, empirical measures of fitness can be wrong because the model used to fit the data is in error. This paper is organized along this dichotomy. The first section in Results reviews the existing analytical model developed to calculate fitness (the theoretical phenotype-fitness map). Then (in ‘An Amended Model’), the existing model is extended to include greater realism; the new results are both analytical and numerical. The third Results section identifies a problem in the direct measurement of fitness. The fourth section revisits the T7 example in light of this new understanding.

## Results

### The Basic Model

The basic model describes the growth rate of a large population of phage, all of the same genotype. The process is thus one of dynamics, not evolution. With constant cell density (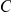), adsorption rate (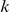), burst size (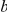), and a strictly invariant lysis time (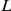), we may write the rate of change of phage density (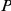) as
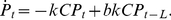
(1)The subscript 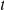 in 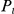 indicates that phage density is a function of time 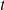. 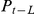 is the phage density 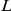 time units in the past. This equation assumes a closed environment and ignores phage loss from death, washout, and all other causes except infection – assumptions appropriate for lab propagation. The equation also assumes phage density is sufficiently low that cell density is not reduced by infection.

An easy solution applies under steady-state conditions, when growth of the phage population becomes exponential. Under exponential growth, we can write 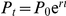 and 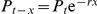, with 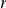 the solution to
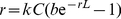
(2)
[5], [6]. The quantity 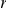 is commonly referred to as the intrinsic growth rate. Although (2) is transcendental, it is easily solved numerically for a given set of parameters.

### An Amended Model

#### A general approach to variation in lysis time: the 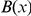 function

**Figure 1:**
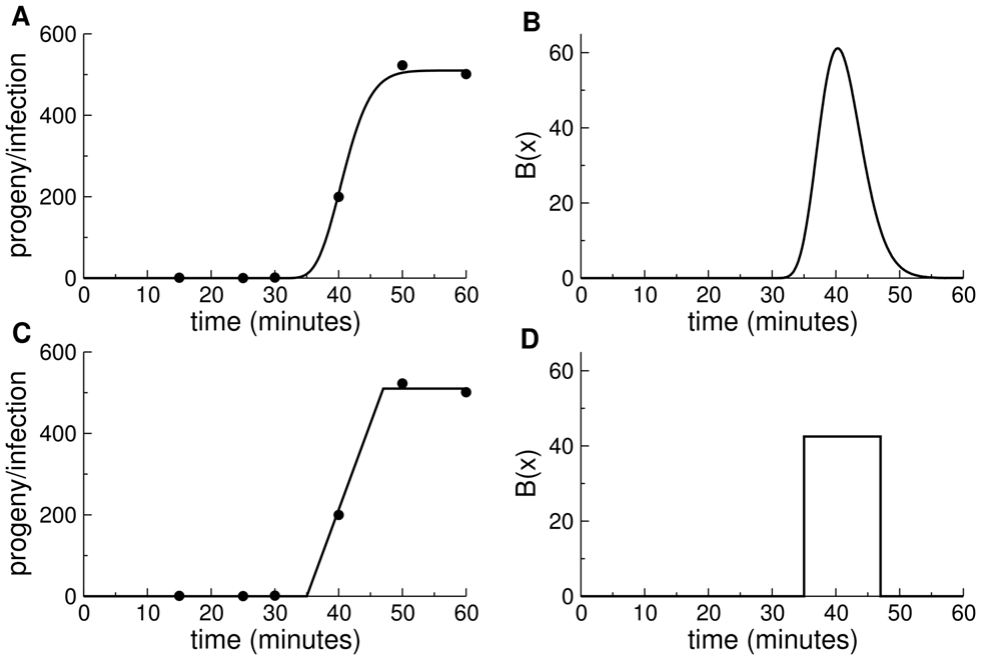
The burst function 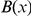 and its derivation from a 1-step growth curve. (A) A sample one-step growth curve [the six data points are from Fig. 4 in Bull et al.([20])]. The curve was fit to a gamma cumulative density function, with appropriate scaling and translation of the time axis by 28 minutes and adjusted for the time of dilution, similar to the method in Heineman et al. [21]. (B) The 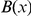 function derived from the curve in (A). The area under the 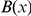 curve equals the height reached by the curve in (A), which is the burst size. (C) The one-step growth data from (A) fit by eye to a straight line. (D) The uniform (rectangular) 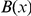 function derived from the function on the left. The two examples merely show the different shapes of 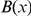 functions that can provide a suitable fit to a one-step assay, although a greater density of data in the assay may not be so permissive of functional forms. Many of the simulations presented below assume the rectangular shape, as in (D).


Although the assumption of fixed lysis time is a sufficiently accurate approximation for many phages, it is violated for many others and is violated even for mutants and novel growth environments of some phages that otherwise show little variance in lysis time. Burst size is highly variable, presumably across all phages [11]. Variance in lysis time and burst size are easily accommodated in (1) above:
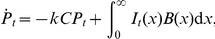


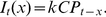
(3)



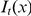
 denotes infections that occurred 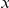 time units before time 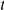. 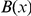 is the burst size function, the rate of progeny production, per cell, of cells infected 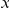 time units in the past, accounting for the possibility that only a subset of infected cells will lyse after 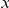 units. 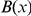 is independent of the state of the culture and hence of time 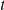. The actual number of progeny released from the average infected cell from 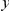 to 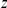 minutes post infection is 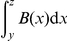 and the average burst size of an infected cell is 
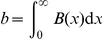
. 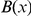 will be zero for times less than some threshold, because infected cells require a minimum time after infection to assemble the first viable progeny (known as the eclipse period, 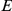). When there is no variance in lysis time, 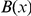 is simply a spike (delta function).


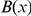
 is found as the derivative of the function of phage titer over time obtained from a standard, one-step growth curve. A one-step curve provides the time course since infection of progeny produced per infected cell, rising from 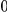 and reaching an asymptote at the burst size (Fig. 1). (In a strict sense, a one-step curve used for deriving 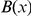 must be corrected for any asynchronous infection.)

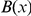
 can be thought of as a composite function derived from separate lysis time and burst size functions. Yet although it is possible to derive 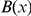 from separate functions of burst size (conditioned on lysis time) and lysis probability over time, it is not possible to work backward from 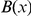 to obtain separate lysis and burst functions unless burst size is independent of 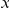 (in which case, the shape of 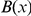 matches the shape of the lysis function). Thus, if 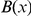 is used to calculate the mean, variance, or other moments of lysis time, those moments will be weighted by any systematic change in burst size with 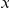. Since 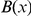 gives the average number of progeny produced per unit time, it does not provide information about variance in burst size among cells lysing at the same time; that variance is in fact irrelevant to the growth rate equations because of the mass action assumption, but is relevant to the fate of genotypes when rare [6].

#### An analytical solution for the amended model

Assuming the population has attained exponential growth, equation (3) can be solved analytically by using the aforementioned fact that 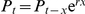. Then 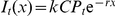 and
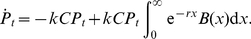
(4)If we rewrite 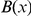 as 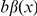, where 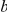 is the average, total burst size, then 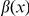 can be treated as a probability density function (pdf) of burst. If the constant 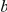 is moved outside, the resulting integral is merely the moment generating function of 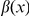, with the dummy variable set to 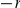. Writing again 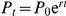, we can simplify (4) to
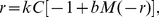
(5)where 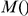 is the moment generating function of 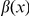.

This decomposition of 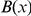 requires that burst size be independent of 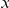. If average burst size changes with 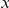, a scaling function must be included inside the integral.

#### Special cases

Given that no progeny can be produced prior to some minimum time after infection (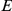), greater flexibility in fitting the model may be offered by modifying the time scale of progeny production. In particular, we may let 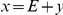 and confine the burst pdf to the interval 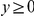. Assuming that 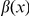 is a gamma pdf, the solution for 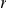 obeys
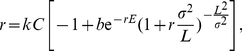
(6)where 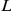 is the mean lysis time and 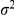 the variance in lysis time (measured on 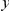). If instead 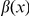 is uniform on 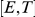, as in Fig. 1D, the solution obeys
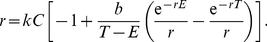
(7)


#### Extensions of the amended model

Two additional modifications of (3) may be needed for realism in serial transfer. One is to include changes in cell density, which can increase from cell division and decrease from infection:
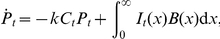


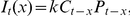


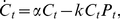
(8)where cell density 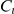 is now a function of time 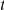 and 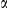 is the growth rate of cells. The fluctuations in cell density will preclude the strict attainment of exponential phage growth, so the previous analytical results will be only approximate [6]. The importance of accounting for changes in cell density can be evaluated by simulation.

A second dimension of realism is to allow variance in the adsorption rate, 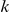. Here, we do not model this effect, because we have no evidence that it is relevant in T7, at least over timescales that should dominate serial transfer dynamics. However, we acknowledge that it might be necessary to include this effect for some systems. There are different ways that nature might vary adsorption rate, and only some are amenable to easy inclusion. One type of variation is to suppose that each burst produces an array of progeny with different adsorption rates, as would result if some virions had 6 tail fibers, others 5, and so on. With this type of variation, each phage particle has a specific adsorption rate that it maintains for life, and the model requires a separate phage equation for each adsorption rate category, all bursts contributing to each category. The cell equation likewise needs a separate loss term for each phage type. Another form of adsorption rate variation is due to phage aging. If adsorption rates decline with time, separate equations for each adsorption rate category are also needed, but one phage type decays into another phage type, rather than all types arising within single bursts. Finally, phage particles may undergo maturation after release, so that their adsorption rate increases with time [12].

### Simulations: effect of lysis time variance and cell growth

A simulation of equations (8) revealed the effects of various elaborations of (1) (Table 1). Results were contrasted between the basic model (fixed 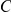 and 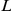) and the amended model with variation in lysis time (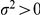) and cell growth (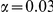, a doubling every 23 minutes, which approximates our system). The 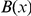 function was either a spike spanning 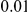 minutes (the minimum interval possible under the numerical integration scheme) or, for the 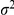 column, was a rectangular function like that in Fig. 1 D. For the 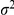 calculation in each row of this table, the base and height of the rectangular 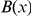 was chosen to achieve the indicated lysis time variance while maintaining the same burst size and average lysis time (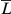) used in the other two columns of that row. The simulation results for the basic model column were found to match analytical results for equations (2) [variance in 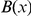 near zero]; likewise, simulation results for the 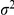 column (large variance) matched (7).

**Table 1:** Simulated phage growth rate per parameter modifications.

	parameters			calculated fitness (dbl/hr)		
Label	k (mL/min)	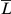 (min)	burst	basic	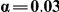	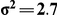
L1	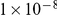	10.0	250	44.2	44.8	47.2
L2	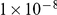	25.0	250	18.4	18.7	18.7
L3	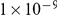	10.0	250	34.0	35.7	35.6
L4	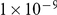	25.0	250	15.6	16.4	15.7
L5	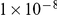	10.0	100	36.8	37.4	38.9
L6	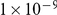	25.0	100	12.8	13.6	12.9

Initial cell density was set at 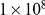 cells/mL. In the basic model, cell density was constant and 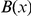 was a spike lasting the minimum interval of iteration (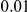 minutes) as an approximation to zero variance. In the 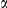 column, 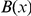 was the same as in the basic model, but cells increased in density at rate 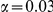 per min. In the 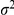 column, the 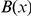 function was rectangular, spanning 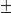
2.85 minutes on each side of the mean so that the variance in lysis time was 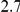 min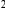; cell density was constant. As a check of the simulation for the basic and 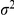 models, fitness estimates were found to be indistinguishable from those in eqns (2) and (7) for their respective cases. In the simulations, densities were updated every 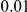 minute.

#### Cell growth (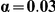)

Allowing cells to grow during single cultures increased the fitness estimate, provided that phage densities were maintained orders of magnitude below cell densities. This qualitative result is obvious for the same reason that phage fitness increases with higher initial cell density, all else equal: higher cell density reduces generation time by reducing the time a phage spends in the free state. The magnitude of the effect seen in Table 1 is not so easily understood, however. It must first be appreciated that the simulation mimicked actual serial transfer in using a dilution and transfer schedule similar to those used in practice: the dilution factor was between 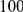 and 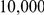, and transfer interval between 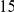 and 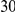 minutes, so that phage densities remained low relative to cell densities. Thus rapid transfer must be used when fitness is high, or the phage will quickly outnumber cells (e.g., 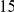 minute transfers are required when fitness is 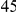 doublings/hr). Since each new culture starts with the same initial cell density, there is less time for cell growth when transfers are rapid than when they are slow. So a larger effect of cell growth is expected for phages with low growth rates because those cultures provide more time for cells to grow before transfer.

In addition, however, the effect of cell growth is larger for phages with low adsorption rates. This counter-intuitive effect of adsorption rate may stem from the fact that the duration of a phage's life that is spent in the free state varies according to the inverse of the product of adsorption rate and cell density (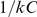). When the product is high, phages spend little time in the free state, so further increases in cell density do not appreciably shorten the free state; when the product is low, further increases in cell density do appreciably shorten the free state, and thus shorten generation time. Thus the benefit of higher cell density stemming from culture growth is realized more at low adsorption rates than at high ones.

#### Variable lysis time (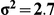)

Allowing variation in 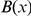 invariably increased fitness, even though the variation was symmetric around the mean lysis time. The reason for this effect is that early bursts contribute more to fitness than late bursts detract. The benefit of variance in lysis time was greater with higher baseline fitness, but this effect may be a trivial consequence of differences in mean lysis time. All trials in this column used the same burst function relative to the mean lysis time, so the early bursts shortened generation time proportionally more when lysis time was short than when long. We also studied the effect of an asymmetric 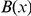 function, but fitness effects could not be attributed solely to skew because other moments varied when skew was changed.

### Estimating fitness directly from sequential titers

Sampling phage titers temporally seems to provide the most relevant and convenient method of measuring fitness directly. Typically, one adds phage to a bacterial culture, transfers a small volume to a new culture at the appropriate time and repeats the process as long as needed. Titers used in the fitness calculation are taken from the culture at the time of transfer to a new culture, the sample being treated with chloroform to kill the cells and stop phage growth. The first sample for the fitness calculation is typically taken after 30 minutes or more of phage growth (which is not usually the first culture in the series), and the second sample is taken an hour or more after the first sample. The growth rate is determined by the difference in titers between the two samplings, adjusted for the time and any transfer dilutions between them. The only issue with this approach is to ensure that the population is growing exponentially. Attainment of exponential growth rests on the population having attained a stable age-of-infection distribution (henceforth “stable age distribution”). A stable age distribution implies that the fraction of infected cells that has been infected for just 1 minute, just 2 minutes, and so on does not change into the future.

**Figure 2:**
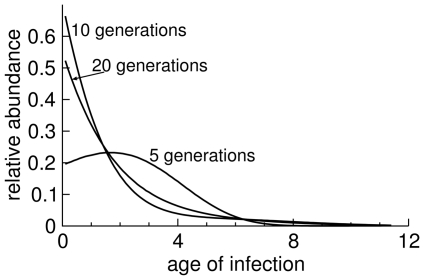
Deviations from stable age distribution in simulations. Each of the three curves shows the profile of infection ‘ages’ at a specific stage of a culture, given as the approximate number of generations indicated (the number of generations associated with each curve is the number of transfers on 12 minute intervals). The 20-generations curve shows the approximate stable age distribution. Curves from earlier times indicate progressively greater deviations from stable age distribution. Infections were simulated as in Table 1. Parameters were chosen to match T7 values: the adsorption rate constant was 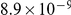 mL/min, cell density was 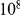 (assumed constant), 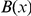 was uniform across 9.3 to 11.5 minutes with a total burst size of 266.

When conducting an assay, the age distribution is clearly not stable at first, because phage are added all at once to a culture of cells. Most infections from those phage will occur rapidly, and then there will be a long interval before the infected cells burst. During this interval, few new infections are occurring. If lysis time is largely invariant, bursts will then also be highly synchronous, and the asynchrony of infection times will persist for many generations. Subtle deviations from a stable age distribution can persist for generations (Fig. 2).

On an intuitive level, we may think of a stable age distribution as represented by a large variance of infection times in the culture relative to the duration of the phage life cycle. Starting from a newly inoculated culture, the variance of infection times (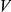) comes both from the variance in lysis time and the variance in time to adsorption. Under exponential decay of free phage from infection, the variance of infection times is 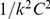; the variance in lysis time depends on 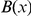. With multiple rounds of infection through time, the variance in infection times will increase linearly (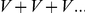) to a first approximation. Thus, one can obtain a qualitative sense of how quickly a stable age distribution will be attained by knowing both the lysis time variance and the loss to adsorption relative to average lysis time.

**Figure 3:**
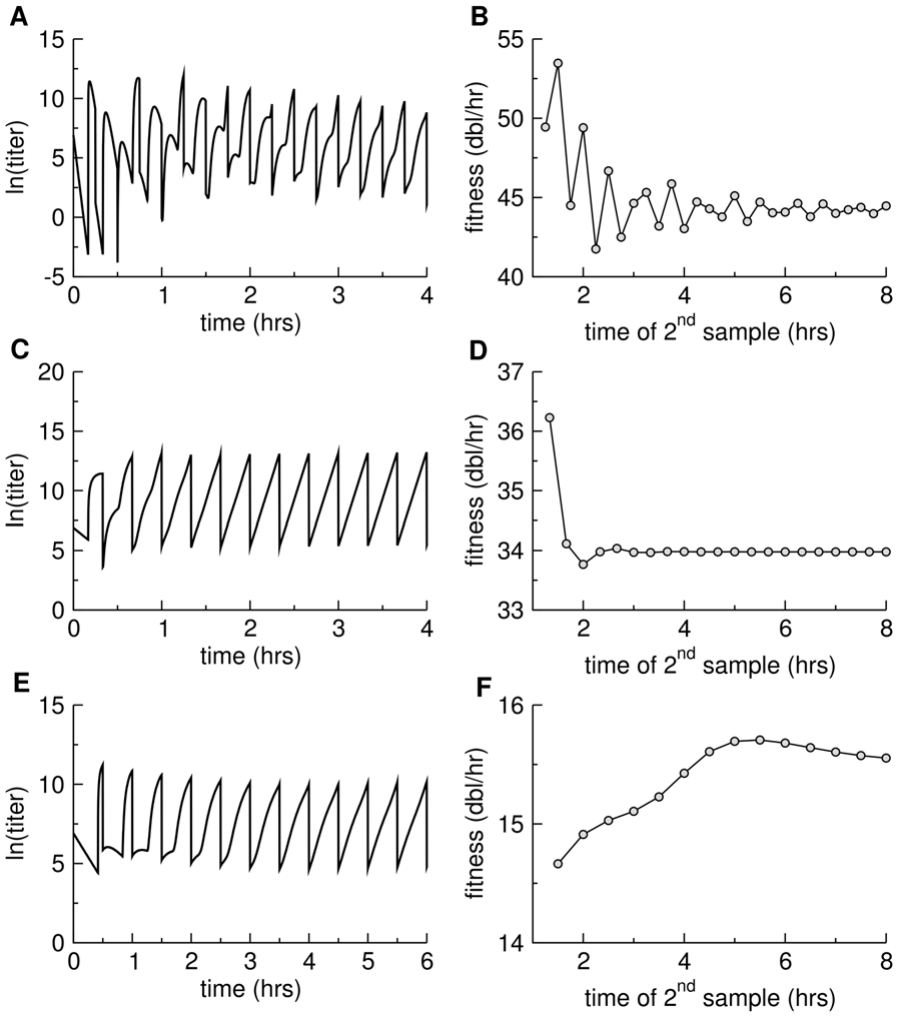
Deviations from stable age distribution in simulated serial transfer protocols and their effects on direct fitness estimation. (A), (C), and (E) each show free phage density as a function of time. Transfers were performed every 15 min (top row), 20 min (middle row) or 30 min (bottom row), the vertical lines indicating the drop in titer following each transfer. When a stable age distribution has been achieved, the set of points from a single culture (between inoculation and transfer) should lie on a straight, inclined line, as is approximately true for the right-most profiles. The absence of stable age distribution is most pronounced at the left end of each figure. (B), (D), and (F) show fitness estimates (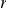) for the respective panels to their left, calculated from titers separated by 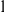 hour. The horizontal axis indicates the time of the second sample used to estimate 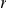. Lines connect the points for ease of visualization but have no specific meaning. The three data sets reveal different patterns of error in fitness estimation, but two sets reveal errors lasting hours after the assay is initiated. Top row is based on conditions L1 from Table 1, basic model; middle row is based on conditions L3, basic model; bottom row is based on conditions L4, basic model.

To illustrate the error introduced by fitting phage titers from two time points, the simulation model of the previous section was run for various conditions and is illustrated for three cases: one with a high adsorption rate and short lysis time, one with a low adsorption rate and short lysis time, and one with a low adsorption rate and long lysis time (L1, L3, and L4, basic model, Table 1). Strong effects of deviation from stable age distribution are seen in all runs, with moderate to large effects on fitness estimation across the first few hours (Fig. 3). The pattern of deviation differs radically between the three conditions, precluding any obvious empirical generalization.

### Revisiting T7

The previous section offered possible reasons for a discrepancy between fitness calculated from the basic model and fitness estimated directly from sequential titers of a serial transfer experiment. We now return to the discrepancy between the two methods of fitness determination specifically for the strain referred to as T7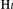 in [8]. Cell density at the time of phage addition was intended to be 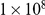/mL. The estimated lysis time was 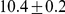 min, burst size was 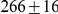 phage, and adsorption rate was 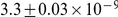 mL/min. With a delta function for 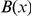, the calculated fitness is 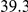 doublings/hr, whereas the directly estimated value is 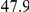.

For the present study, we considered the possibility that some of the prior parameter estimates were in error. Fitness calculations are highly sensitive to lysis time and adsorption rates because of the effect on generation time (less so for burst size), so lysis time and adsorption rates were re-evaluated. There was no suggestion that lysis time was different from that reported, but adsorption rate was re-estimated as 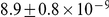, a 2.7-fold increase. We also checked for the possibility of adsorption rate variation when measured over different intervals; there was no evidence that the adsorption rate constant measured over two minutes differed from that over 4 minutes, and no evidence of an effect of aging the lysate for 24 hr before the assay; thus we use a model of fixed 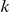.

The fitness calculated from the basic model for these parameters is 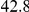, the increase over 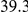 due to the higher adsorption rate. The calculation rises modestly to 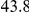 by including cell growth (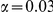, a doubling time of 23 min) and variance in lysis time (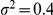, as per [8], using the 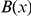 function of Table 1). To illustrate the sensitivity of calculated fitness to lysis time, the estimate of 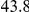 rises to 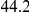 merely by decreasing mean lysis time 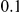 minute to 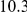, all else equal. It is not practical to estimate mean lysis time to the nearest 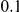 minute, thus the calculated fitness cannot be precise.

**Figure 4:**
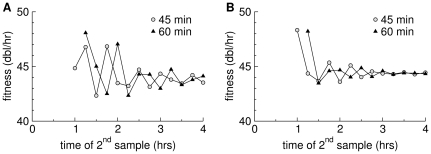
Fitness estimates in a simulation of T7 infection during serial transfer every 15 minutes. Estimates are based on samples separated by 60 minutes or 45 minutes, as indicated. Parameters are from the text and Fig. 2 for T7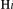, except that the variance is 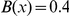 in (A) and 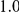 in (B), using a rectangular 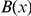 function as in Fig. 1D. The oscillations are stronger with the smaller variance in lysis time. The horizontal axis shows the time at which the second titer was taken. The estimates shown use data only from the second simulated culture onward.

The direct fitness estimate could be affected by the failure of the culture to obtain a stable age distribution. An inflated direct estimate would help bring the two numbers into agreement. A simulation shows that the oscillations in the estimate of 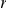 are potentially large enough to account for the high reported value (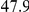) if the variance of 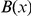 is as small as reported, but not if it is slightly larger (Fig. 4).

**Figure 5:**
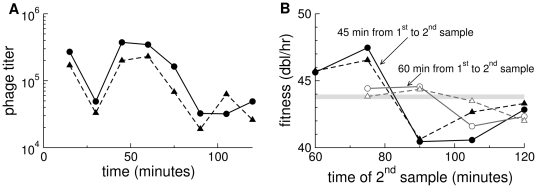
Violation of stable age distribution in laboratory transfers of T7 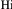. (A) Sequential titers in two independent cultures. Inter-transfer intervals (15 min) and transfer volumes (5 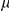
L of 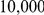) were the same for all transfers, so the common titer oscillation out to 90 min presumably reflects a violation of stable age distribution. (B) Calculations of 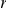 from these data based on titers spanning 60 minutes (45 minutes) vary, without converging before 120 min for the second sample. The horizontal axis refers to the time at which the second sample was taken; the time of the first sample depends on whether the estimate spanned 45 or 60 minutes. The horizontal, gray bar is the approximate value of the fitness calculated in this paper from the phenotype-fitness map parameterized with burst size, lysis time, and adsorption rate estimates.

To assess the possibility of deviations from stable age distribution, we performed new serial transfers of T7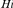, this time recording phage titer at each transfer out to 2 hr; fitness estimates in the previous study were based on titers taken at 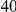 min and at 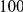 min. Methods were as before [8], except that transfers this time were made every 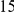 min instead of every 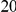 min. Sequential titers reveal a behavior characteristic of the failure to have attained stable age distribution (Fig. 5), and calculations of 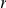 based on these data fail to stabilize before 2 hr. Importantly, these new data suggest a direct fitness estimate of 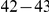 that is compatible with the calculated one and considerably lower than the published one.

At face value, there is reconciliation between the former directly estimated fitness of T7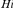 and the current one (the previous one was plausibly inflated by a violation of stable age distribution); there is also agreement between the calculated and directly estimated fitnesses provided here. Some qualifications are warranted, however. First, it is not strictly possible to revisit the former fitness estimate; frozen cell stocks were not the same for the two sets of assays, and the ingredients used for LB broth now and then possibly differ (LB is not a defined medium). Thus, conditions used in the assays are subject to unknown variation. Second, the reconciliation is likely no better than approximate, because the simulations show that fitnesses are sensitive to deviations in parameter values that lie well within measurement error. Third, manual serial transfer assays are cumbersome and do not provide the level of resolution needed for a detailed resolution of within-culture dynamics. A more complete resolution of the empirical dimensions of this problem thus awaits automation of serial transfer and a reporter system to monitor infection rates in real time.

The temporal pattern of 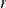 values calculated from simulations parameterized with T7 data in Fig. 4 does not match the empirical pattern, at least in the nature of the oscillations. The basis for this discrepancy is not clear, but there are some obvious possibilities to consider. First, as shown in Fig. 3, the temporal pattern is qualitatively sensitive to at least modest variations in individual parameters. It is thus plausible that combinations of T7 parameters statistically compatible with the observed values could be found that lead to a better fit of the 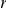 calculations. More fundamentally, however, the biological titers differ from the simulated titers. Simulated titers are of free phage. The titers estimated in an experiment are taken from a sample of the culture treated with chloroform and thus include free phage and phage released by cells partway through infection when exposed to chloroform. It is challenging to model this reality: the number of phage released from an infected cell depends on the number of progeny produced since infection and on how many of those progeny are released from the cell by chloroform treatment. The effect of chloroform is not easily calculated because chloroform merely creates lesions in cells but does not lyse them unless the infection has proceeded long enough that lysin has accumulated inside the cell.

## Discussion

This study provides the most accurate model to date of the phenotype-fitness map of bacteriophage growth. This map applies specifically to the idealized environment of serial propagation on a continual excess of hosts. The model developed here extends a previous analytical solution by allowing distributions of lysis time (rather than a fixed time) and in using simulation to measure sources of error in fitness measurement. Although the specifics apply to bacteriophage growth, some of the principles are likely to be general. In particular, a deviation from stable age distribution and the use of full parameter distributions rather than parameter means is likely of broad relevance to fitness measures in other experimental microbial systems.

The model developed here is dynamic rather than evolutionary, but its chief utility is evolutionary prediction. By specifying phage fitness as a function of three parameters (or parameter distributions), the model is easily used to understand how fitness changes with changes in the parameters, hence how selection operates on each of the three phenotypes. The immediate value of such a model is to improve our ability to apply experimental studies of phages in testing evolutionary models and in interpreting laboratory evolution of phages and other viruses. Such experimental systems often offer the only real hope of testing models intended for viruses relevant to human health, so the correct interpretation of phage studies is important. More broadly, work such as this enhances an appreciation of the strengths and limitations of phenotype-fitness maps of other organisms.

For the parameter combinations considered here, the cumulative effect of errors can easily lead to a 5% difference between measured and calculated fitness, and even a 10% error is feasible. As growth rate is a geometric measure of fitness, any error compounds rapidly over generations. However, with an awareness of the possible problems, it is also feasible to anticipate and perhaps reduce errors by including appropriate extensions of the basic model, with simulations if necessary. Application of this new perspective to T7 data seemingly explained the large discrepancy between the two methods of fitness estimation evident in prior work. It should also be appreciated that, even with this new understanding, the methods of parameter estimation are too coarse to provide accuracy beyond 1–2 doublings/hr when phage fitness is as high as in T7.

As noted, a large potential error arises in the direct calculation of an exponential growth rate if the culture has not neared a stable age-of-infection distribution. The deviation from stable age distribution decays with time, but the rate of decay depends highly on the variance in lysis time and adsorption rate in the culture. Thus the extent to which deviation from a stable age distribution is a problem will vary from phage to phage and also vary with culture conditions. Various empirical methods to hasten the approach to stable age distribution may be attempted. For example, reducing the concentration of cells in the initial culture has the effect of slowing infections and thus of dispersing them over time, compared to a culture with high cell density. Thus it might be supposed that a more rapid approach to stable age distribution could be achieved with a protocol in which the first culture used low cell density. Yet this protocol does not appear to work well (simulations not shown), as it leads to a high density of free phage in the first culture, and those free phage are quickly adsorbed when transfered to a culture with high cell density. An alternative protocol might be to inoculate phage incrementally into the first culture, spreading their infections over time (suggested by a reviewer). We have not evaluated this approach, but as with any attempt to bypass natural decay, it runs the risk of delaying decay instead of hastening it; such a protocol might need to be tailored specifically to each system.

The existence of large estimation errors has implications for the interpretation of some phage studies. One is that the measurement of small fitness effects must be interpreted with caution, at least when those fitnesses constitute a form of growth rate [13]–[16]. Small fitness effects may be real for the exact measurement assay, but the present study suggests that small effects measured in one assay may not translate across even minor variations in protocol. Use of competition strains to estimate a relative fitness may alleviate some of the problem, but if the two strains in the same culture experience different approaches to stable age distribution, then even competitor strains will face the problem identified here.

In many studies, errors in fitness estimation may have little impact. For example, the optimal lysis time evolves as 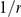 [5]. To first order, a small percent error in 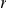 leads to the same percent error in 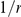. Thus, if 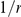 is 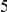 minutes, a 10% error is 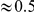 minutes, barely detectable in lysis time assays, and potentially much smaller than the deviation between the predicted optimum and the observed endpoint of evolution (as in [8]). Much of phage experimental evolution is about large magnitudes of evolution, and the types of errors identified here will not change conclusions based on those effects.

Our analysis underscores that mapping specific phenotypes into fitness is complicated, and that predicted fitness values are highly sensitive to details of individual phenotypes (e.g., the exact lysis time). These findings are reminiscent of results by Wahl and coauthors [6], [17], [18], who found that fixation probabilities can differ widely for rare mutations affecting different phenotypes, even if these mutations have the same effect on long-term growth rate (i.e., fitness). They also found that fixation probabilities were dramatically altered by the exact timing of dilutions [6]. In summary, theirs and our results show that mapping phenotypes to fitness and evolutionary success is a difficult and non-trivial undertaking, even in a system as simple as bacteriophage grown in a chemostat.

An obvious, if difficult next step is to model the intracellular determinants of the three phage parameters of burst size, lysis time, and adsorption rate. This deeper model would provide the basis for understanding the evolution of intracellular mechanics through their effects on the higher-level phenotypes. This level of understanding appears within reach: Endy et al. [19] generated a kinetic simulation model of the life cycle of phage T7, with over 100 genetic elements and based entirely on empirically determined parameter values. As methods improve for measuring intracellular concentrations and localization of phage proteins, we may be able to move to this next level and create a molecular-based phenotype-fitness map.

## Methods

Assays of sequential titers of phage T7 used the serial transfer methods of Heineman and Bull [8]. Briefly,*E. coli* K-12 cells (strain IJ1133) were grown in 10 mL of LB broth in 125 mL flasks in an orbital waterbath with agitation for 1 hr at 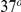 C to a density of 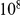 cells/mL. Phage were added, grown for 15 min, and a sample of the culture was transfered to another culture of cells that had been incubating for 1 hr. A sample of the completed flask was extracted over chloroform and used for titer determination. Phage titers were maintained two orders of magnitude below cell densities to ensure that host density was only negligibly depressed by infection.

Adsorption assays were conducted by adding phage to a culture of cells that had been incubated for 1 hr to a density of 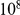 cells/mL (following the same protocol used for sequential titer measures). Cell density (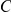) was measured just prior to phage addition by plating to determine colony forming units. At time 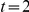 (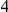) minutes after phage addition, a sample was removed. Total phage titer (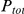) was determined by plating an aliquot immediately, and free phage titer (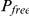) was determined by plating an aliquot of the supernatant of a centrifuged culture (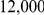 rpm for 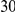 sec). The adsorption rate constant (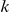) was estimated from
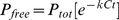
(9)in units mL/min. This ‘end-point’ determination runs the risk of underestimating 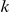 when 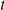 is large (S. Abedon, pers. comm.), but should be largely free of this problem for the time intervals used here.

Numerical analyses of phage population dynamics (equations (8)) were used for Figs. 3 and 4 and Table 1. Computer programs to conduct these analyses were written in C, with variables updated every 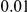 minute.
